# 

**Published:** 1912-11

**Authors:** 


### CONTENTS.

ORIGINAL COMMUNICATIONS:—

TTreatment of Osophageal Stricture. By George C. Mizell, Atlanta, Ga. ........ 407

The Functions of a Medical Milk Milk Commission. By Clarence Rhodes, M. D., Atlanta, Ga. ........ 412

Five Cases of Pellagra Treated With Gelsemium. By Roy Blosser, M. D., Atlanta, Ga. ........ 423

EDITORIAL ........ 427

SELECTIONS AND ABSTRACTS ........ 429

MEDICAL ITEMS ........ 454

